# The Effects of Combined Social Cognition and Interaction Training and Paliperidone on Early-Onset Schizophrenia

**DOI:** 10.3389/fpsyt.2020.525492

**Published:** 2020-09-30

**Authors:** Yichen Li, Ke Sun, Denghua Liu, Mo-Xian Chen, Guo Li, Jun Ma, Xiaofan Zhang

**Affiliations:** ^1^ Department of Child & Adolescent Psychiatry, Wuhan Mental Health Center, Tongji Medical College, Huazhong University of Science & Technology, Wuhan, China; ^2^ Department of Psychiatry, Tongji Hospital, Tongji Medical College, Huazhong University of Science & Technology, Wuhan, China; ^3^ School of Rehabilitation, Kunming Medical University, Kunming, China

**Keywords:** social cognition and interaction training, cognition, social functioning, symptomatology, early-onset schizophrenia

## Abstract

**Background:**

The limitations associated with antipsychotics in early-onset schizophrenia patients have stimulated more interest in psychological interventions in this population. Nevertheless, the isolated psychosocial interventions are unrealistic in a treatment success covering the complex array of symptoms, and the psychosocial interventions could be an adjunct treatment to the pharmacological treatment. It is necessary to find the benefits of psychological interventions with limited and targeted use of antipsychotics. Social cognition and interaction training (SCIT) was a program for social cognitive rehabilitation in adult schizophrenia. However, it is unclear how generalizable this is to early-onset patients.

**Methods:**

The current study tested this hypothesis that combined SCIT and paliperidone was superior to paliperidone alone in treating early-onset schizophrenia patients on cognitive, functional, and symptom outcomes. Two hundred eight inpatients with schizophrenia aged 13 to 17 years old participated in a 24-week work intervention program. Patients completed a battery of measures administered at a pre-SCIT intervention baseline, 4, 8, 12, and 24 weeks post-SCIT, respectively.

**Results:**

SCIT had significant added benefits above paliperidone for the speed of processing, attention/vigilance, and social cognition of the Chinese version of MATRICS consensus cognitive battery (MCCB) domains (p<0.05). The following logistic regression analysis on the exploration of the influential factors also confirmed the effects of SCIT. However, combined SCIT and paliperidone intervention had a null impact on social functioning and symptomatology.

**Conclusions:**

The present study provides the first evidence that combined SCIT and paliperidone intervention has the potential to improve cognitive functions for the early-onset schizophrenia patients. The findings in the current study are suggestive of the extreme importance of SCIT as an adjunctive treatment in early-onset schizophrenia patients.

## Introduction

Schizophrenia is a chronic, severe, and disabling illness that affects approximately 1% of individuals in the population, with onset usually during late adolescence or early adulthood (1). Youth who experience early-onset schizophrenia, defined by an onset before 18 years of age, often have impaired cognition involving both neuro-cognition and social cognition (2–4). These deficits highly correlated with loss of function in school, community, and social relationships (5, 6). As cognitive impairments remain an enduring and functionally disabling symptom of schizophrenia, it needs to find treatments to combat the cognitive deficits in younger persons with this condition (7).

Early-onset schizophrenia defined by an onset strictly before the age of 18 years (8). Patients with early-onset schizophrenia were less responsive to pharmacological treatment on cognitive deficits (9, 10). Furthermore, antipsychotics often have severe metabolic, neurological, and other side effects associated with significant premature mortality (11). The limitations associated with antipsychotic medication in early-onset schizophrenia patients have stimulated greater interest in psychological interventions in this population (12). Some aspects of social cognition had indicated to predict functional outcomes, and mediate the relationship between neuro-cognition and functional outcome in schizophrenia patients (13–15). The promise of social skills training focused on social cognition has been documented in schizophrenia patients (16).

Social cognition and interaction training (SCIT), which designed to increase the flexibility in attributional style and improve metacognition, is comprised of teaching individuals problem identification, goal setting, use of role-plays or behavioral rehearsal for successful social interaction, social modeling, and problem-solving (17). Contrary to other psychosocial interventions that focused on the specific social cognitive impairments to the exclusion of other domains (18), SCIT targets a broader range of social cognition, e.g., a theory of mind, social perception, social knowledge, attributional bias, and emotion processing (19). It indicated as a program for social cognitive rehabilitation in adult schizophrenia (20).

There is clear evidence that SCIT is a helpful intervention on social cognition in adults with schizophrenia (21), but it is unclear how generalizable this is to the early-onset patients. Such group of patients may have more severe social cognitive deficits, negatively influencing social, cognitive and psychological development (22), and having a worse prognosis and outcome (23, 24), which if alleviated might change the course of the disorder (25). Therefore, it is needed to confirm the degree to which SCIT is promising in youth with schizophrenia. Although delusions and catatonic symptoms occur less frequently, early-onset schizophrenia patients usually have hallucinations, thought disorder, and flattened affect. The isolated psychosocial interventions are unrealistic in a treatment success covering the complex array of symptoms, and the psychosocial interventions could be an adjunct treatment to the pharmacological interventions (26). The China Food and Drug Administration approved paliperidone extended-release for the treatments of adolescents with schizophrenia in 2017. Therefore, the specific objective of the current study was to examine the efficacy of combined SCIT and paliperidone on early-onset schizophrenia patients with a short duration of illness, the entire course of disease included previous episodes is no more than 4 years.

Since social cognition mediates the relationship between neuro-cognition and functional outcome, and negative symptoms also have a strong relationship with social cognitions (27), we expect that the combined SCIT and paliperidone improve cognitions along with social functioning and the symptomatology in the early-onset schizophrenia patients. In the current study, we used the MATRICS Consensus Cognitive Battery (MCCB) to estimate the patients’ cognition functions; the personal and social performance scale (PSP) to measure the social functioning; the scale for the assessment of Negative Symptoms (SANS) and the positive and negative syndrome scale (PANSS, total score) to assess the symptomatology. The current study hypothesized that combined SCIT and paliperidone were superior to paliperidone alone in treating early-onset schizophrenia patients on cognitive, functional, and symptom outcomes. This study will assist practitioners in understanding the psychosocial interventions with medications in early-onset schizophrenia patients.

## Materials and Methods

### Participants

Two hundred and eight inpatients with schizophrenia aged 13 to 17 years at the time of the study, who met the tenth version of the International Classification of Diseases (ICD-10) criteria for schizophrenia, were recruited from the Wuhan Mental Health Center, Tongji Medical College of Huazhong University of Science& Technology. Enrolled criteria were (1) meeting criteria for schizophrenia according to ICD-10; (2) no evidence of current or previous head injury, CNS disease, and other ICD-10 disorders; (3) right-handed with normal vision and hearing; (4) no history of antipsychotic use, or more than 2 weeks from the last drug treatment, and no long-acting injection of antipsychotic drugs. The excluded criteria were (1) with acute suicidal ideation or life-threatening, (2) severe, or unstable physical disorder; (3) had a history of substance or alcohol use disorder within six months before screening; (4) had an estimated IQ<70, as assessed by the Wechsler Intelligence Scale (Chinese version). Participants, to whom the doctor in charge deemed inappropriate for any other reason, were also excluded from the study. During the study intervention periods, some patients proceeded from wards to back home by the improvement of symptomatology, but they still need oral medication and rehabilitation training. All the participants under the investigation had the written and informed consent from the guardian. The ethics committee of the Wuhan Mental Health Centre, Tongji Medical College of Huazhong University of Science& Technology, approved the study protocol and informed consent procedures (KY20161617).

### Study Design

**Figure 1:**
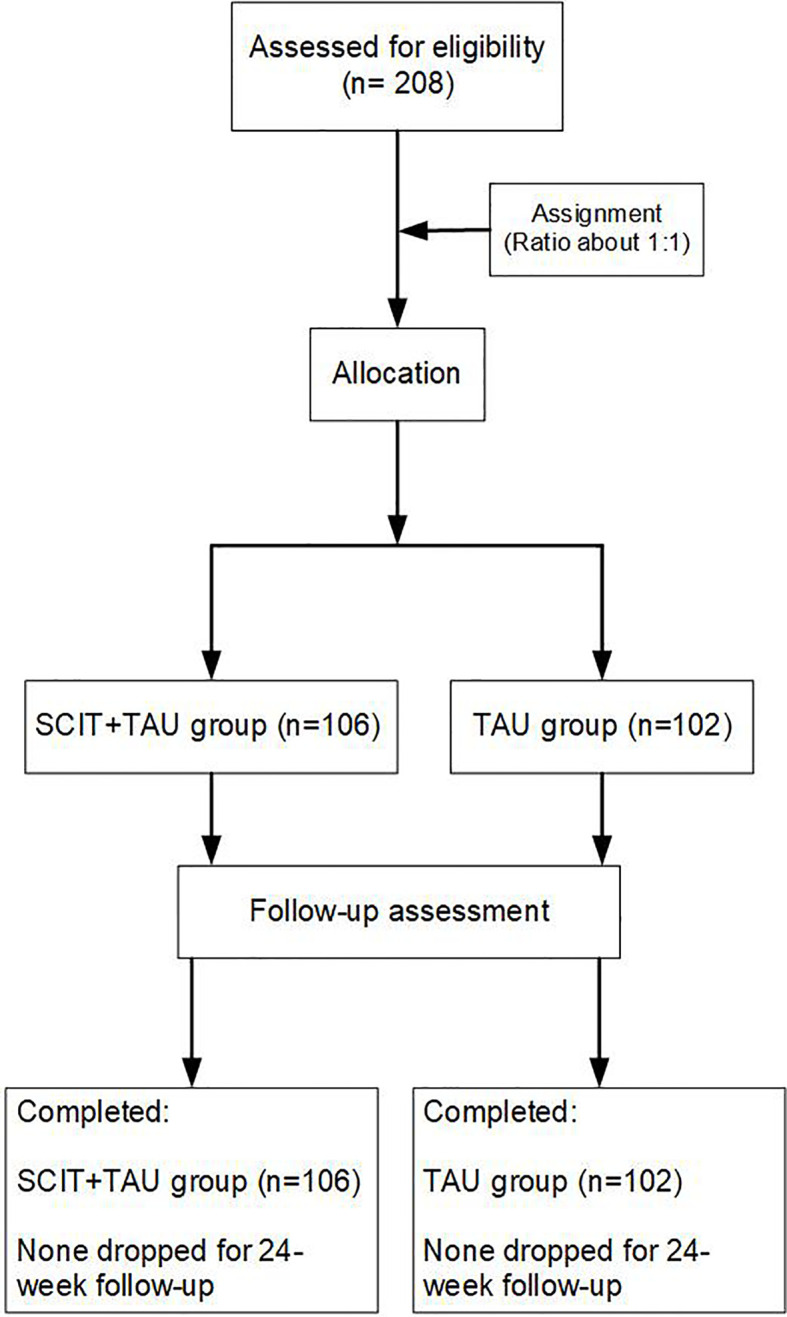
The CONSORT flow diagram.

Patients screened for inclusion and exclusion criteria were randomized to either of the two groups on a 1:1 ratio. Patients who received SCIT plus paliperidone (paliperidone extended-release tablets, INVEGA^®^, 3 to 12 mg/day, Xian Janssen, Co. Ltd) enrolled in the SCIT+ paliperidone group (n=106). Those received paliperidone only were in the paliperidone group (n=102). More details are shown in **Figure 1**. Patients in SCIT plus paliperidone intervention were delivered for 5-8 patients at a time by a pair of clinicians, who trained to use SCIT by the developers of the intervention. There are 15 sub-groups in the SCIT+ paliperidone intervention group. Group leaders were psychologists and occupational therapists. Those in the groups received a 45- to 60-min training course once per week for 20 to 24 sessions. Patients’ social functioning and cognitive function were assessed by the personal and social performance scale (PSP) and the Chinese version of MATRICS consensus cognitive battery (MCCB) respectively; the scale measured their symptom severity for the assessment of Negative Symptoms (SANS) and the positive and negative syndrome scale (PANSS, total score). Patients completed measures of SANS, PANSS, and PSP at a pre-SCIT intervention baseline, 4, 8, 12, and 24 weeks post-SCIT, respectively, and MCCB at baseline and 24 weeks post-SCIT. Patients in the paliperidone group completed the same tests at the same time as those in the SCIT+ paliperidone group. The raters were well trained and had excellent interrater reliability; the raters were blind to the randomization.

### Statistical Analysis

The sample size was calculated by PASS software 15.0.5. We used Pearson’s Chi test for the screening of the differences in gender ratio, education, dependant’s type, dependants’ education, family relationship, and relapse frequency between groups. One-way analysis of variance (ANOVA) performed for the analysis of each continuous variables between groups, such as age, paliperidone dosage, duration of the illness, SANS, PANSS and PSP at baseline and MCCB at baseline(Speed of processing, Attention/vigilance, Working memory, Verbal learning, Visual learning, reasoning and problem-solving, and Social cognition). The changes of MCCB, SANS, PANSS, and PSP were estimated by repeated-measures ANOVA, respectively, with the group (SCIT+ paliperidone vs paliperidone) as a between-group factor and time point (baseline vs follow-up) as a within-group factor.

To clarify the effects of SCIT on the early-onset schizophrenia patients, we performed binary logistic regression analysis to explore the potentially influential factors for improved cognition, social functioning, and symptoms. The logistic regression model is routinely used to evaluate the treatment effect on the responder status at the first time point of interest corresponding to the end of the study. The odds ratio means the character of the treatment effect adjusted for baseline. It was calculated by the Exp(β) in the SPSS software.

In the regression analysis, the full samples subdivided according to the median value of the variables identified as screening factors. The variables with *p* < 0.2 in the independence test (Chi-square and ANOVA tests) submitted to the regression analysis as the covariates, the effect sizes of MCCB cognition domains, SANS, PANSS total score, and PSP were the dependent respectively in the separate binary logistic regressions for the screening progress. The effect size calculated as (score at 24 weeks score at baseline)/pooled SD of baseline.

## Results

### Sociodemographic and Clinical Profiles of Participants

Two hundred and eight patients completed the study, 106 of them were in the SCIT+ paliperidone group, and 102 of them were in the paliperidone group. The demographic characteristics of the participants were in **Table 1**. Patients in the SCIT plus paliperidone group had not much significant difference from those in the paliperidone group, except for the higher paliperidone dosage in the paliperidone group (*p*<0.05, **Table 1**).

**Table 1:** Patients’ sociodemographic and clinical profiles.

Variables	SCIT+ paliperidone	Paliperidone	Test statistic
Gender			χ*^2^* = 0.19, *p* = 0.67
Male (*n*; %)	54 (50.94)	55 (53.92)	
Female (*n*; %)	52 (49.06)	47 (46.08)	
Age(years *± SD*)	16.11 ± 1.44	16.13 ± 1.43	*F* = 0.01, *p* = 0.94
Education (*n*; %)			χ*^2^* = 1.76, *p* = 0.42
6 years	8 (7.55)	8 (7.84)	
9 years	48 (45.28)	55 (53.92)	
12 years	50 (47.17)	39 (38.24)	
Dependants type (*n*; %)			χ*^2^* = 1.58, *p* = 0.45
Grandparents	16 (15.10)	20 (19.61)	
Single-parent	42 (39.62)	44 (43.14)	
Two-parent	48 (45.28)	38 (37.25)	
Dependants’ education (*n*; %)			χ*^2^* = 3.74, *p* = 0.29
6 years	25 (23.58)	15 (14.71)	
9 years	25 (23.58)	32 (31.37)	
12 years	38 (35.85)	34 (33.33)	
16 years	18 (16.99)	21 (20.59)	
Family relationship (*n*; %)			χ*^2^* = 2.42, *p* = 0.12
Harmony	52 (49.06)	61 (59.80)	
Conflict	54 (50.94)	41 (40.20)	
Relapse frequency			χ*^2^* = 0.65, *p* = 0.89
0	35 (33.02)	29 (28.43)	
1	45 (42.45)	45 (44.12)	
2	23 (21.70)	24 (23.53)	
3	3 (2.83)	4 (3.92)	
Paliperidone dosage (mg/day*±* SD)	9.37 ± 2.98	10.41 ± 2.28	*F* = 4.35, *p* = 0.04*
Duration of the illness (months*±* SD)	12.39 ± 10.14	13.19 ± 10.354	*F*= 0.32, *p* = 0.57
MCCB at baseline (mean *±* SD)			
Speed of processing	29.11 ± 5.46	27.85 ± 5.41	*F* = 2.80, *p* = 0.10
Attention/vigilance	33.41 ± 5.61	33.10 ± 5.50	*F* = 0.16, *p* = 0.69
Working memory	34.33 ± 5.13	33.80 ± 6.14	*F* = 0.45, *p* = 0.50
Verbal learning	34.69 ± 5.60	33.89 ± 6.43	*F* = 0.91, *p* = 0.34
Visual learning	34.44 ± 5.07	34.01 ± 5.88	*F* = 0.33, *p* = 0.57
Reasoning and problem solving	35.86 ± 4.43	36.25 ± 4.75	*F* = 0.39, *p* = 0.53
Social cognition	35.36 ± 4.84	34.15 ± 5.35	*F* = 2.94, *p* = 0.09
SANS scores at baseline (mean *±* SD)	17.33 ± 2.632	17.02 ± 2.53	*F* = 0.75, *p* = 0.39
PANSS—total scores at baseline (mean *±* SD)	62.44 ± 4.90	62.49 ± 4.54	*F *= 0.01, *p *= 0.94
PSP scores at baseline (mean *±* SD)	50.22 ± 6.46	50.66 ± 6.91	*F* = 2.23, *p* = 0.64

Significance levels included in the p value, *p < 0.05.

### Effects of SCIT+ Paliperidone in the Change of Cognition, Social Functioning, and Symptom Severity

**Figure 2:**
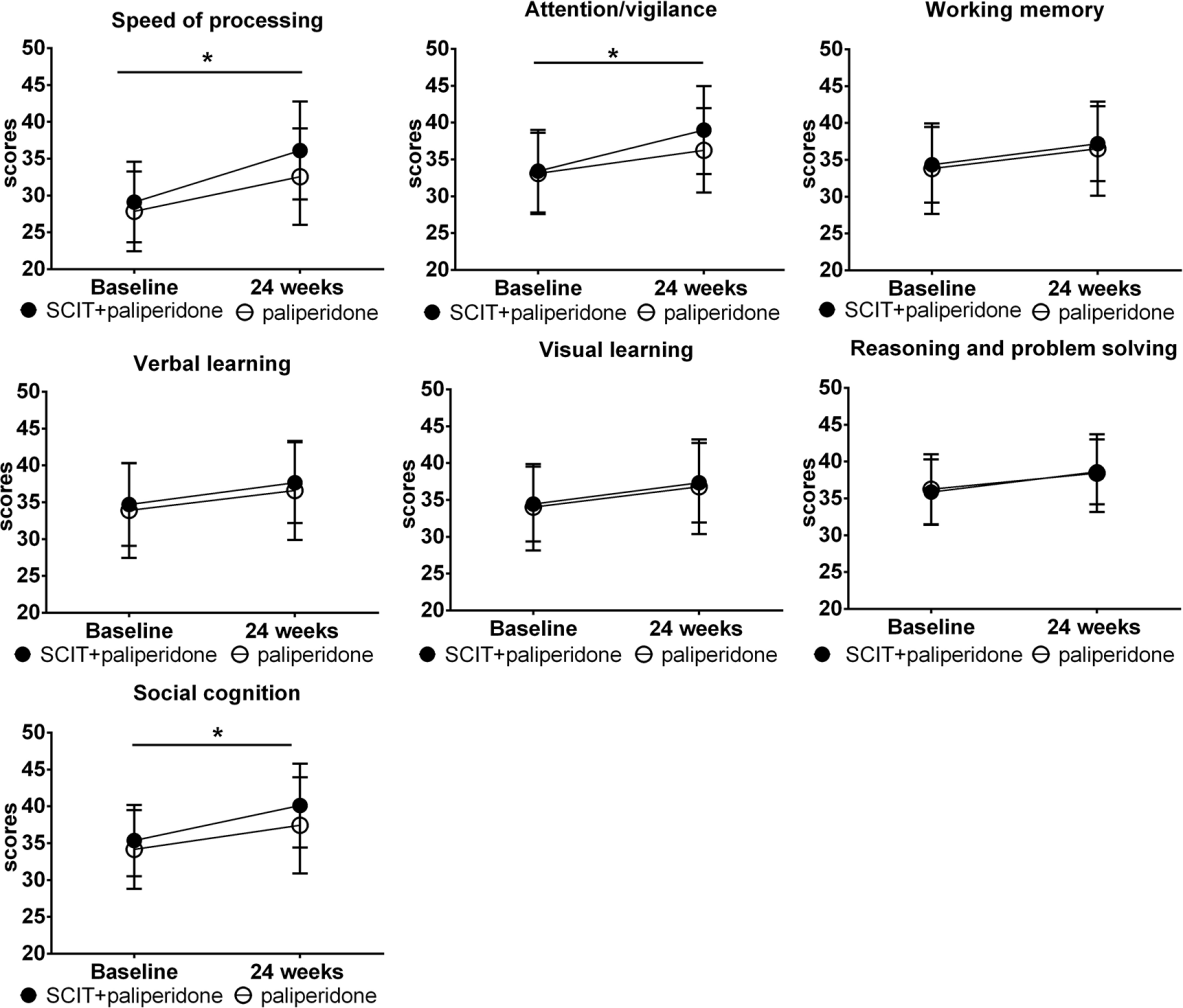
The comparison of changes in the scores of MCCB cognitive domains from baseline to the 24-week endpoint. Data are presented as mean ± SD. Significance levels are included in the p-value, *P < 0.05.

**Figure 3:**
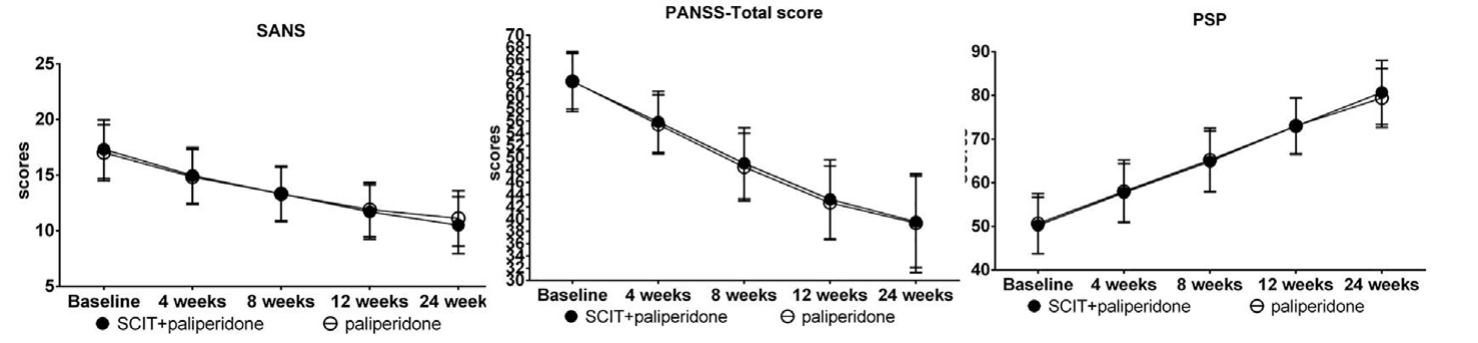
Comparison of changes in the scores of SANS, PANSS, and PSP from baseline to 4-week interim timepoint, 8-week interim timepoint, 12-week interim time point, and 24-week endpoint. Data are presented as mean ± SD.

All the patients completed the 24-week study. During the study intervention periods, 85 patients in the SCIT+ paliperidone group and 79 patients in the paliperidone group proceeded from wards to back home. They still had routine outpatient visiting every once a week. In the MCCB test, the repeated-measures one-way ANOVA revealed a significant main effect of group for the speed of processing, attention/vigilance, and social cognition (*p*<0.05). Generally, both groups of treatments improved all the MCCB cognitive domains after 24-week intervention, but the SCIT+ paliperidone group had more sound effects than paliperidone group on the above domains (**Table 2**, **Figure 2**). Similarly, SANS and PANSS decreased, but PSP increased after 24 weeks of intervention in both groups. However, repeated-measures one-way ANOVA did not find significant differences between SCIT+ paliperidone and paliperidone group (**Figure 3**).

**Table 2:** Results of repeated ANOVA analysis on the measures of cognition, social functioning, and symptom severity.

	df	F	*p*-value	Cohen’s *d*
MCCB domain				
Speed of processing				
Group (SCIT+ paliperidone vs. paliperidone)	1,206	9.47	0.00*	0.86
Attention/vigilance				
Group (SCIT+ paliperidone vs. paliperidone)	1,206	3.91	0.04*	0.71
Working memory				
Group (SCIT+ paliperidone vs. paliperidone)	1,206	0.60	0.44	0.48
Verbal learning				
Group (SCIT+ paliperidone vs. paliperidone)	1,206	1.27	0.26	0.45
Visual learning				
Group (SCIT+ paliperidone vs. paliperidone)	1,206	0.40	0.53	0.48
Reasoning and problem solving				
Group (SCIT+ paliperidone vs. paliperidone)	1,206	0.03	0.86	0.51
Social cognition				
Group (SCIT+ paliperidone vs. paliperidone)	1,206	6.60	0.01*	0.67
SANS scores				
Group (SCIT+ paliperidone vs. paliperidone)	1,206	0.05	0.82	-1.56
PANSS—total scores				
Group (SCIT+ paliperidone vs. paliperidone)	1,206	0.28	0.60	-1.75
PSP scores				
Group (SCIT+ paliperidone vs. paliperidone)	1,206	0.00	0.99	1.81

Significance levels are included in the p-value, *p < 0.05.

### Possible Influential Factors for the Improved Cognition, Social Functioning, and Symptoms

The results of logistic regression analysis also confirmed the effect of SCIT+ paliperidone. In the MCCB cognition domain, the multivariate analysis (binary logistic regression model) revealed that the speed of processing was associated with the speed of processing at baseline, attention/vigilance at baseline, working memory at baseline and group difference (SCIT+ paliperidone vs paliperidone). The maximum effect was in the SCIT+ paliperidone group. Patients in that group had a 2.89-folder higher chance to obtain improved speed of processing. Attention/vigilance was associated with the working memory at baseline, verbal learning at baseline, and group; the patients in the SCIT+ paliperidone group had a 6.38-folder higher chance to obtain improvement. Social cognition was also associated with visual learning at baseline, social cognition at baseline, and group; the patients in the SCIT+ paliperidone group had a 3.15-folder higher chance to obtain improvement. SANS was also associated with SANS at baseline, PSP at baseline, and group, patients in the SCIT+paliperidone group, had a 53.3% greater chance to obtain improvement. More other results in the logistic regression analysis displayed in **Supplementary Table 1**.

## Discussion

In this study, we compared the early-onset schizophrenia patients’ performances in the SCIT plus paliperidone intervention with those in the only paliperidone intervention. None of the participants dropped out throughout the whole program, which suggested that SCIT and paliperidone appear to be feasible and well tolerated by patients with early-onset schizophrenia. Patients who received SCIT + paliperidone showed significant improvement in the speed of processing, attention/vigilance, and social cognition of MCCB domains relative to those with the only paliperidone. Those domains of neuro-cognition refers to the processes of linking and appraising information (28), which is a better predictor of functioning than the positive symptoms in schizophrenia patients (14). Those deficits have been associated with impairments in different domains of functional outcome such as global psychosocial functioning (29), community functioning (30), daily problem solving skills (31), job tenure (32), social skills (33), or independent living skills (34). However, results indicated that SCIT had no added benefits above paliperidone for enhancing the social functioning and clinical symptoms in the patients with early-onset schizophrenia.

We chose MCCB to estimate patients’ cognition, as it serves as the standard measure for cognitive studies, includes social cognition as one of the seven cognitive domains in schizophrenia (35). MCCB evidenced sensitivity to cognitive differences between adolescent healthy controls and adolescents with early-onset schizophrenia (36). The repeated-measures one-way ANOVA had found the significant differences in the change of the speed of processing, attention/vigilance, and social cognition in patients between the SCIT + paliperidone and paliperidone group. The following logistic regression analysis on the exploration of the influential factors also confirmed the effects of SCIT. Notably, most of them have the largest OR value, which means SCIT has a substantial impact on the improvements. Researchers have noted that the speed of processing and attention/vigilance in the MCCB was associated with the motivation (37). The accurate processing of socio emotive stimuli intimately integrated with neural systems related to motivation in the human brain (38), deficits in processing social stimuli harm motivation in patients with schizophrenia. Through this mechanism, it showed a negative influence on their functioning (39). SCIT could improve motivation in adult schizophrenia patients (40). Therefore, we suspect that the effects of SCIT on the cognitions measured by MCCB are due to its direct impact on the motivation of the early-onset schizophrenia patients.

We failed to find the similar effects of SCIT on the MCCB domains of working memory, verbal learning, visual learning, reasoning and problem-solving. The tasks of the above MCCB domains were possibly dependent on the individuals’ self-ability of perception, which impaired in the baseline of the study. SCIT has been reported to be effective in improving the ability to interact with social cues (41), but not emotion perception abilities to oneself (42).

The personal and social performance scale (PSP) scale is a reliable and valid instrument for assessing the social functioning of patients with schizophrenia during treatment (43). We used PSP to assess the patients’ social functioning from four main domains, such as socially useful activities, personal and social relationships, self-care, and disturbing and aggressive behavior. SCIT had no significant added benefits above paliperidone for facilitating remission of the social functioning. Previous studies had reported the social behaviors in the milieu had the most active associations with verbal learning, visual learning and memory, and reasoning and problem solving (14). However, neither of those MCCB domains obtained significant improvements in the SCIT plus paliperidone group compared with those in the paliperidone group. SCIT has reported improving the PSP scores in Chinese adult schizophrenia patients (44).

Nevertheless, we failed to find similar effects on the youth with schizophrenia. PSP has been proposed as particularly well sensitive to assess the social functioning outcomes in antipsychotic drug (45). Therefore, we suspected that the power of paliperidone might cover the net impacts of SCIT on the social functioning of early-onset schizophrenia patients.

Similarly, SCIT plus paliperidone intervention had null effects on the symptomatology of the early-onset schizophrenia patients. The lack of additive effects of SCIT on perception and executive functioning in the current study cannot help early-onset schizophrenia patients to detect social cues and understand the intentions while interacting with others, which in turn has no progress on negative and positive symptoms in schizophrenia (14). Moreover, The PANSS total score at baseline in our study was about 62.44 ± 4.903, which means those patients had mildly ill with higher function (46). SCIT has thought to have less impact on higher functioning patients (47). A relative more significant impact on social cognition than on social functioning and negative symptoms would be promising as SCIT improves social cognition through a combination of rehearsal-based “bottom-up” learning and acquisition strategies.

The early-onset schizophrenia patients usually mean less independence, more inadequate education (22); those also thought to influence the SCIT treatment effects. On the contrary, the logistic regression analysis in the current study revealed the impact of SCIT on early-onset patients was independent with those background characters, except for the cognition, social functioning, and symptomatology at the baseline. Moreover, there were more than 200 patients enrolled in this study; the sample size was large enough to have the power of avoiding a type II error. All of those have shown SCIT is feasible for use with and well-tolerated by early-onset schizophrenia patients in real-world settings.

The current study has several limitations. First, most of the patients in the present study were first-episode or have once relapse with the mean age of 16 years old, which increases the risk of diagnosis uncertainty, such as bipolar disorder in the future. Second, we only studied the treatment effect between baseline and 24-week interim assessments; we are not clear that those effects will last or change for a long time. Third, the SCIT acts as role-play with more than one facilitator, which was conducted by themselves (48), which has linked to several facets, including the theory of mind, emotion perception, and empathic ability (49). Therefore, there was an inherent probability for the children to understand the purpose of the training. Last, there is no active control in the current study, all the patients received paliperidone treatments, and we had not controlled for the dosages. However, research on the eﬀects of antipsychotic medication on cognition is inconsistent, and pediatric psychotic disorders are scant. There is evidence that new atypical antipsychotic medications may have some positive eﬀect on cognitive functioning, but there is no evidence that antipsychotic medication completely ameliorates cognitive impairments. Future study with a long-term follow-up and the more complicated situation is warranted.

In summary, the present study provides the first evidence that SCIT+ paliperidone intervention has the potential to improve cognitive functions for the early-onset schizophrenia patients. Our primary findings indicate that SCIT as an adjunctive treatment may facilitate remission of the schizophrenia symptoms in youth. Those changes could improve the long-term prognosis and psychosocial outcomes, which may also prevent some of the secondary difficulties associated with schizophrenia from being accrued, e.g., interruptions in education, reliance on welfare benefits, downwards socio-economic shift, and breakdown of relationships. This study could help to find how psychological interventions should be adapted for children and adolescents, the benefits of psychological interventions with limited and targeted use of antipsychotics, and the most effective timing for interventions during illness. Our study is the first report on a large number of young people with a relatively short duration of schizophrenia receiving individual cognitive remediation treatment early in their psychiatric history in China. The findings in the current study are suggestive of the extreme importance of social cognition training in early-onset schizophrenia patients.

## Data Availability Statement

All datasets generated for this study are included in the article/supplementary material.

## Ethics Statement

The studies involving human participants were reviewed and approved by The ethics committee of the Wuhan Mental Health Centre, Tongji Medical College of Huazhong University of Science & Technology (KY20161617). Written informed consent to participate in this study was provided by the participants’ legal guardian/next of kin.

## Author Contributions

JM and XZ were involved in the conceptualization and planning of the study. YL, KS, and DL were involved in the implementation of the trial. M-XC and XZ were responsible for the management of the trial, including the implementation of randomization procedures, data management, and drafting the manuscript. GL was responsible for the statistical analyses. All authors contributed to the article and approved the submitted version.

## Funding

This work was financially supported by the critical project in the Wuhan health and Family Planning Commission to YL (grant WX17B15).

## Conflict of Interest

The authors declare that the research was conducted in the absence of any commercial or financial relationships that could be construed as a potential conflict of interest.
