# Supplementary material for: The Effects of Combined Social Cognition and Interaction Training and Paliperidone on Early-Onset Schizophrenia
Source: Front Psychiatry. 2020 Sep 30;11:525492. doi: 10.3389/fpsyt.2020.525492 (PMC7556232; doi:10.3389/fpsyt.2020.525492)
Supplement: Supplementary file 1 [file Table_1.docx]

Supplementary Material

## Supplementary Table1

Supplementary Table1 Final binary logistic regression models for each effect sizes according to the sociodemographic and clinical profiles

|  |  | B | S.E. | Wald | df | P | OR | 95% CI |
| --- | --- | --- | --- | --- | --- | --- | --- | --- |
| Effect size of speed of processing (MCCB cognition domain) | | | | | | | | |
| Speed of processing at baseline | ≤28 | -0.98 | 0.35 | 7.98 | 1 | 0.01 | 0.38 | 0.19-0.74 |
|  | >28 |  |  |  |  |  |  |  |
| Attention/vigilance at baseline | ≤33 | 0.89 | 0.34 | 6.72 | 1 | 0.01 | 2.43 | 1.24-4.75 |
|  | >33 |  |  |  |  |  |  |  |
| Working memory at baseline | ≤34 | 0.86 | 0.30 | 8.02 | 1 | 0.01 | 2.36 | 1.30-4.27 |
|  | >34 |  |  |  |  |  |  |  |
| Group | paliperidone | 1.06 | 0.31 | 12.06 | 1 | 0.00 | 2.89 | 1.59-5.26 |
|  | SCIT+ paliperidone |  |  |  |  |  |  |  |
| Constant | | -1.10 | 0.33 | 11.18 | 1 | 0.00 | 0.33 |  |
| Effect size of attention/vigilance (MCCB cognition domain) | | | | | | | | |
| Working memory at baseline | ≤34 | 0.79 | 0.33 | 5.61 | 1 | 0.02 | 2.20 | 1.15-4.21 |
|  | >34 |  |  |  |  |  |  |  |
| Verbal learning at baseline | ≤35 | -0.98 | 0.34 | 8.50 | 1 | 0.00 | 0.38 | 0.20-0.73 |
|  | >35 |  |  |  |  |  |  |  |
| Group | paliperidone | 1.85 | 0.33 | 31.04 | 1 | 0.00 | 6.38 | 3.32-12.25 |
|  | SCIT+ paliperidone |  |  |  |  |  |  |  |
| Constant | | -1.35 | 0.32 | 17.89 | 1 | 0.01 | 0.26 |  |
| Effect size of working memory (MCCB cognition domain) | | | | | | | | |
| Visual learning at baseline | ≤35 | -1.06 | 0.32 | 11.21 | 1 | 0.00 | 0.35 | 0.19-0.65 |
|  | >35 |  |  |  |  |  |  |  |
| Social cognition at baseline | ≤35 | -0.66 | 0.32 | 4.42 | 1 | 0.04 | 0.52 | 0.28-0.96 |
|  | >35 |  |  |  |  |  |  |  |
| Constant | | 0.80 | 0.23 | 12.02 | 1 | 0.00 | 2.23 |  |
| Effect size of verbal learning (MCCB cognition domain) | | | | | | | | |
| Working memory at baseline | ≤34 | -1.27 | 0.36 | 12.74 | 1 | 0.00 | 0.28 | 0.14-0.56 |
|  | >34 |  |  |  |  |  |  |  |
| Verbal learning at baseline | ≤35 | -1.34 | 0.43 | 9.69 | 1 | 0.00 | 0.26 | 0.11-0.61 |
|  | >35 |  |  |  |  |  |  |  |
| Visual learning at baseline | ≤35 | 2.14 | 0.48 | 19.65 | 1 | 0.00 | 8.52 | 3.30-21.97 |
|  | >35 |  |  |  |  |  |  |  |
| SANS at baseline | ≤18 | -0.60 | 0.31 | 3.89 | 1 | 0.05 | 0.55 | 0.301-1.00 |
|  | >18 |  |  |  |  |  |  |  |
| Constant | | 0.21 | 0.27 | 0.60 | 1 | 0.44 | 1.24 |  |
| Effect size of visual learning (MCCB cognition domain) | | | | | | | | |
| Reasoning and problem solving at baseline | ≤36 | 0.91 | 0.29 | 9.95 | 1 | 0.00 | 2.48 | 1.41-4.36 |
|  | >36 |  |  |  |  |  |  |  |
| Constant | | -0.75 | 0.2 | 13.00 | 1 | 0.00 | 0.47 |  |
| Effect size of reasoning and problem solving (MCCB cognition domain) | | | | | | | | |
| Attention/vigilance at baseline | ≤33 | -0.62 | 0.30 | 4.34 | 1 | 0.04 | 0.54 | 0.30-0.96 |
|  | >33 |  |  |  |  |  |  |  |
| Constant | | -0.37 | 0.20 | 3.40 | 1 | 0.07 | 0.69 |  |
| Effect size of social cognition (MCCB cognition domain) | | | | | | | | |
| Visual learning at baseline | ≤35 | -1.17 | 0.38 | 9.66 | 1 | 0.00 | 0.31 | 0.15-0.65 |
|  | >35 |  |  |  |  |  |  |  |
| Social cognition at baseline | ≤35 | 1.68 | 0.38 | 19.92 | 1 | 0.00 | 5.34 | 2.56-11.14 |
|  | >35 |  |  |  |  |  |  |  |
| Group | paliperidone | 1.15 | 0.31 | 13.83 | 1 | 0.00 | 3.15 | 1.72-5.76 |
|  | SCIT+ paliperidone |  |  |  |  |  |  |  |
| Constant | | -0.90 | 0.28 | 10.25 | 1 | 0.00 | 0.41 |  |
| Effect size of SANS | | | | | | | | |
| SANS at baseline | ≤18 | -1.00 | 0.31 | 10.78 | 1 | 0.00 | 0.37 | 0.20-0.67 |
|  | >18 |  |  |  |  |  |  |  |
| PSP at baseline | ≤50 | -0.68 | 0.30 | 5.05 | 1 | 0.03 | 0.51 | 0.28-0.91 |
|  | >50 |  |  |  |  |  |  |  |
| Group | paliperidone | -0.63 | 0.30 | 4.31 | 1 | 0.04 | 0.53 | 0.29-0.97 |
|  | SCIT+ paliperidone |  |  |  |  |  |  |  |
| Constant | | 0.61 | 0.29 | 4.52 | 1 | 0.03 | 1.84 |  |
| Effect size of PANSS Total score | | | | | | | | |
| Visual learning at baseline | ≤35 | -0.83 | 0.33 | 6.50 | 1 | 0.01 | 0.44 | 0.23-0.83 |
|  | >35 |  |  |  |  |  |  |  |
| PANSS total score at baseline | ≤62.5 | -1.23 | 0.33 | 13.67 | 1 | 0.00 | 0.29 | 0.15-0.56 |
|  | >62.5 |  |  |  |  |  |  |  |
| Constant | | -0.12 | 0.30 | 0.16 | 1 | 0.69 | 0.89 |  |
| Effect size of PSP | | | | | | | | |
| SANS at baseline | ≤18 | -0.94 | 0.32 | 8.79 | 1 | 0.00 | 0.39 | 0.21-0.73 |
|  | >18 |  |  |  |  |  |  |  |
| PSP at baseline | ≤50 | -1.90 | 0.32 | 35.24 | 1 | 0.00 | 0.15 | 0.08-0.28 |
|  | >50 |  |  |  |  |  |  |  |
| Constant | | 1.23 | 0.27 | 20.56 | 1 | 0.00 | 3.43 |  |
